# Deletion of either the regulatory gene *ara1* or metabolic gene *xki1* in *Trichoderma reesei* leads to increased CAZyme gene expression on crude plant biomass

**DOI:** 10.1186/s13068-019-1422-y

**Published:** 2019-04-09

**Authors:** Tiziano Benocci, Maria Victoria Aguilar-Pontes, Roland Sándor Kun, Ronnie J. M. Lubbers, Kathleen Lail, Mei Wang, Anna Lipzen, Vivian Ng, Igor V. Grigoriev, Bernhard Seiboth, Paul Daly, Ronald P. de Vries

**Affiliations:** 1 Fungal Physiology, Westerdijk Fungal Biodiversity Institute & Fungal Molecular Physiology, Utrecht University, Uppsalalaan 8, 3584 CT Utrecht, The Netherlands; 2 US Department of Energy Joint Genome Institute, 2800 Mitchell Drive, Walnut Creek, CA 94598 USA; 3 Department of Plant and Microbial Biology, University of California Berkeley, Berkeley, CA 94598 USA; 4 Research Area Biochemical Technology, Institute of Chemical, Environmental and Bioscience Engineering, TU Wien, 1060 Vienna, Austria

**Keywords:** Plant biomass degradation, *Trichoderma reesei*, Xylan, Cellulose, CAZymes, Xyr1

## Abstract

**Background:**

*Trichoderma reesei* is one of the major producers of enzymes for the conversion of plant biomass to sustainable fuels and chemicals. Crude plant biomass can induce the production of CAZymes in *T. reesei*, but there is limited understanding of how the transcriptional response to crude plant biomass is regulated. In addition, it is unknown whether induction on untreated recalcitrant crude plant biomass (with a large diversity of inducers) can be sustained for longer. We investigated the transcriptomic response of *T. reesei* to the two industrial feedstocks, corn stover (CS) and soybean hulls (SBH), over time (4 h, 24 h and 48 h), and its regulatory basis using transcription factor deletion mutants (Δ*xyr1* and Δ*ara1*). We also investigated whether deletion of a xylulokinase gene (Δ*xki1*) from the pentose catabolic pathway that converts potential inducers could lead to increased CAZyme gene expression.

**Results:**

By analyzing the transcriptomic responses using clustering as well as differential and cumulative expression of plant biomass degrading CAZymes, we found that corn stover induced a broader range and higher expression of CAZymes in *T. reesei*, while SBH induced more pectinolytic and mannanolytic transcripts. XYR1 was the major TF regulating CS utilization, likely due to the significant amount of d-xylose in this substrate. In contrast, ARA1 had a stronger effect on SBH utilization, which correlates with a higher abundance of l-arabinose in SBH that activates ARA1. Blocking pentose catabolism by deletion of *xki1* led to higher expression of CAZyme encoding genes on both substrates at later time points. Surprisingly, this was also observed for Δ*ara1* at later time points. Many of these genes were XYR1 regulated, suggesting that inducers for this regulator accumulated over time on both substrates.

**Conclusion:**

Our data demonstrates the complexity of the regulatory system related to plant biomass degradation in *T. reesei* and the effect the feedstock composition has on this. Furthermore, this dataset provides leads to improve the efficiency of a *T. reesei* enzyme cocktail, such as by the choice of substrate or by deleting *xki1* to obtain higher production of plant biomass degrading CAZymes.

**Electronic supplementary material:**

The online version of this article (10.1186/s13068-019-1422-y) contains supplementary material, which is available to authorized users.

## Background

Plant biomass is the most abundant renewable carbon source on Earth for conversion into biofuel and biochemicals by the biotechnology industry. It is composed of three major polysaccharides (cellulose, hemicellulose and pectin), and many different plant biomass degrading (PBD) enzymes are required for efficient degradation [1]. *Trichoderma reesei* (*Hypocrea jecorina*) is a cellulolytic filamentous saprobic fungus that has been used in biotechnology for many decades, mainly for cellulase production [2]. Major developments for industrial applications were reviewed previously [3], such as the improved-cellulase producer QM9414 strain or the hypersecreting RUT C30 strain (CCR derepressed, due to a partial truncation of CRE1) [4]. The disaccharide lactose is the primary industrial inducer of *T. reesei* cellulase production, but induction by crude plant biomass is a promising alternative. However, a challenge remains to sustain induction when the limited inducers that are available have been consumed. Sustaining induction for longer time periods will lead to higher overall CAZyme yields as well as a more diverse array of CAZymes and reduce enzyme production costs.

While *T. reesei* has been suggested as a model to study plant biomass degradation [5], its strategy to degrade plant biomass differs from most other saprobes studied so far, as its genome encodes a narrow set of enzymes, some of which (e.g., several cellulases) are produced at high levels [6]. Its molecular mechanisms for the production of plant biomass degrading enzymes and sugar catabolism have been studied [7–10], demonstrating that XYR1 is the major cellulolytic and xylanolytic regulator [8], but also involved in d-xylose and (partially) l-arabinose catabolism. A second regulator, ARA1, regulates l-arabinose and d-galactose releasing as well as catabolic enzymes in response to l-arabinose and d-galactose [9].

**Fig. 1:**
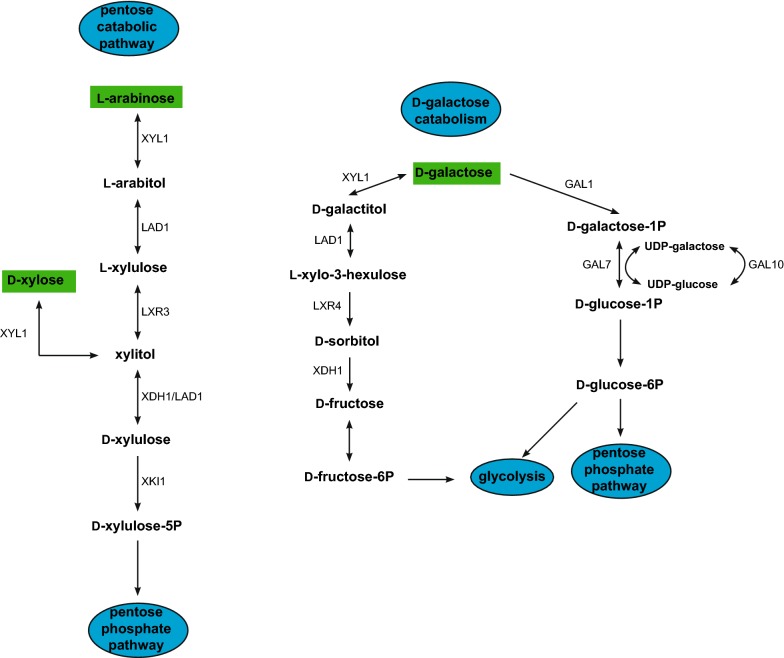
Pentose catabolic pathway and d-galactose oxido-reductive and Leloir catabolic pathways in *T. reesei.* The PCP diagram illustrates where the Δ*xki1* deletion affected the pathway. The d-galactose oxido-reductive pathway illustrates the shared enzymes with the PCP pathway. The diagrams were adapted from [11]

Carbon catabolism related to plant biomass conversion has been studied in many fungi and recently reviewed [11]. Carbon catabolism in *T. reesei* is peculiar, as, more enzymes play a role in multiple catabolic pathways, compared to several other saprobes [11]. For example, the *T. reesei* pentose catabolic pathway (PCP) and the d-galactose oxido-reductive pathway share three enzymes (Fig. 1), and each of these pathways are regulated by both XYR1 and ARA1 [9]. When genes of carbon catabolic pathways are deleted, this can lead to the accumulation of metabolites, which can function as inducers. In *Aspergillus niger*, a xylulokinase mutant (where the last step of the PCP is blocked), resulted in accumulation of inducers, such as xylitol and l-arabitol [12]. In *T. reesei,* xylitol and l-arabitol accumulation was observed in deletion strains (Δ*xyl1*, Δ*lad1* and Δ*lxr3*) of three earlier steps of the PCP [13]. While there are no reports of the effect of a full deletion of the final step of the PCP, the xylulokinase (XKI1), its antisense inhibition increased xylitol production in *T. reesei* [14].

Transcriptomics is a sensitive tool to probe complex regulatory events, but only a few *T. reesei* transcriptomics studies have been performed using crude plant biomass [15–17], while others used polymers or mono- and disaccharides [3]. In particular, only one transcriptomic study analyzed a regulatory mutant (Δ*xyr1*) using crude plant biomass (wheat bran) [18], whereas no catabolic mutants have been analyzed by transcriptomics using crude plant biomass. This previous study, analyzing only a single time point, identified a set of genes regulated by XYR1 (including not only (hemi-)cellulolytic genes, but also genes encoding non-enzymatic cellulose active enzymes, sugar transporters and heat shock proteins) [18]. Analysis across multiple time points is required to uncover the dynamic changes in gene expression patterns as the crude plant biomass is degraded by the fungus.

In this study, we analyzed the transcriptome of *T. reesei* during growth on the two industrial substrates soybean hulls (SBH) and corn stover (CS) over time, using two regulatory mutants (Δ*xyr1* and Δ*ara1*) and one metabolic mutant (Δ*xki1*). These two substrates have different polysaccharide compositions [19, 20], allowing us to deeply explore how the regulatory system responds to a wide array of sugar inducers released from these polysaccharides. CS is richer in hemicellulose, particularly arabinoxylan, while SBH is richer in pectin, xyloglucan and mannan (Additional file 1). This study resulted in three main findings. *T. reesei* had higher and broader transcript levels of PBD CAZyme genes when cultured on CS. ARA1 had a larger role in the regulation of PBD transcripts on SBH compared to XYR1, which was the major TF regulating plant biomass degradation in CS. The block of pentose catabolism (by deletion of *xki1* from the PCP) led to higher PBD CAZyme expression at later time points in the CS and SBH cultures.

## Results

### The regulatory and catabolic mutants had severely reduced growth on pure mono- and polysaccharides compared to the reference strain, but not on crude plant biomass

**Fig. 2:**
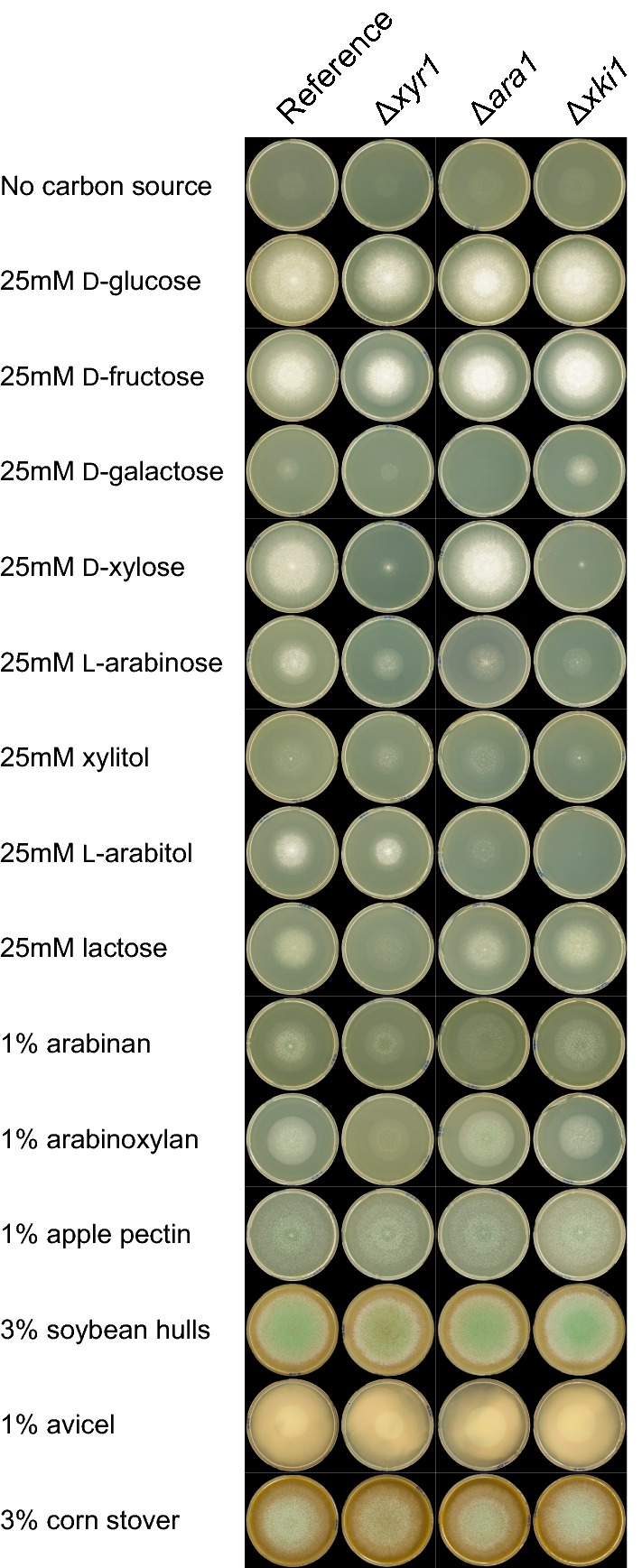
Growth profile of *T. reesei* reference strain (QM9414) and deletion mutants Δ*xyr1*, Δ*ara1* and Δ*xki1* on mono- and disaccharides, polymers and crude plant biomass. All plates were incubated for 5 days at 28 °C. The growth on Avicel of all mutants was comparable with the reference strain

Three *T. reesei* deletion mutants (Δ*xyr1*, Δ*ara1* and Δ*xki1*) were phenotypically compared to the reference strain QM9414 by growth on various carbon sources, including mono- and disaccharides, polymers and crude plant biomass (Fig. 2). As reported previously [21, 22], deletion of *xyr1* most severely affected growth on d-xylose, less severely on lactose and to an even lesser extent on l-arabinose, xylitol, arabinan and arabinoxylan (Fig. 2). Deletion of *ara1* abolished growth on d-galactose and reduced growth on l-arabinose, l-arabitol, while growth on d-xylose, arabinan, arabinoxylan and lactose was not affected (Fig. 2). Deletion of the xylulokinase *xki1* resulted in strongly reduced growth on d-xylose and to a lesser extent on l-arabinose and xylitol, while growth on l-arabitol was abolished (Fig. 2). In all three deletion strains, strong growth reduction was only observed on specific mono- and polysaccharides, but not on crude plant biomass (Fig. 2), perhaps reflecting the diversity of nutrients available in plant biomass and highlighting the complex regulatory network during growth on crude substrates.

### The *T. reesei* reference strain expressed PBD CAZymes at higher levels on corn stover than on soybean hulls

**Fig. 3:**
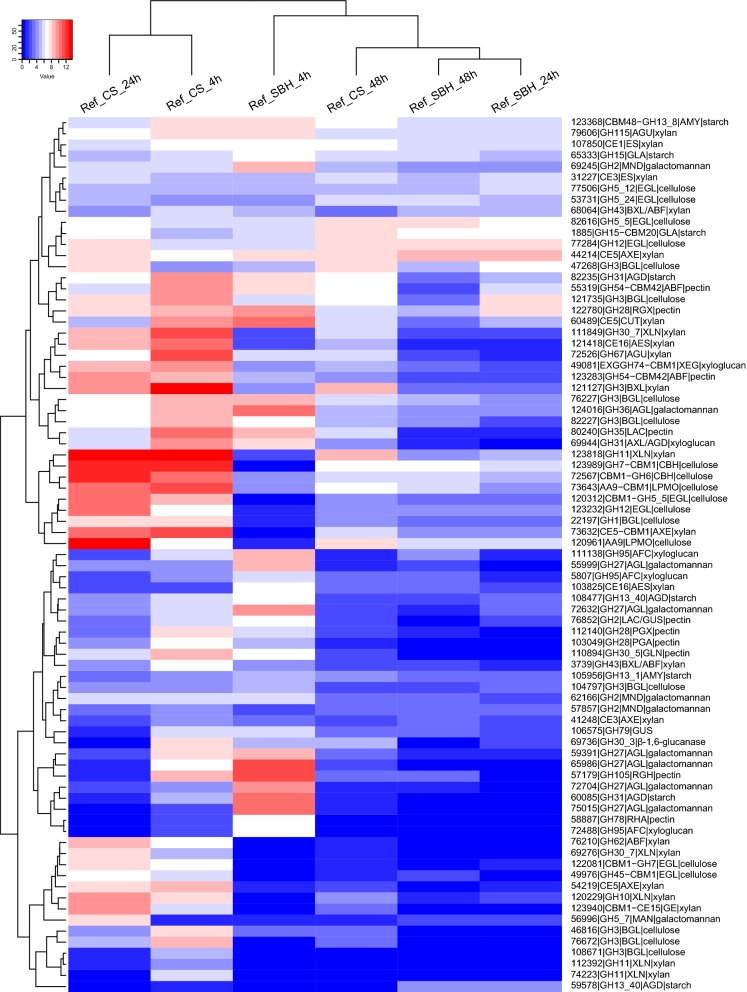
Hierarchical clustering (Euclidean distance) of PBD CAZyme gene expression in the *T. reesei* reference strain on corn stover (CS) and soybean hulls (SBH)

CS or SBH cultures from transfer experiment were sampled after 4, 24 and 48 h for transcriptome analysis that focused on genes encoding PBD CAZymes, carbon catabolic enzymes and related TFs (Additional file 4). To investigate the adaptation to each substrate, we initially compared the PBD CAZyme transcriptome profiles by clustering all six conditions of the reference strain (Fig. 3). The 4 h and 24 h CS samples clearly separated from the other samples, while the 4 h SBH sample was also distant from the remaining samples (Fig. 3). Interestingly, the 24 h and 48 h SBH samples clustered with the 48 h CS sample (Fig. 3), and overall had less highly expressed genes than the other samples. This suggests that during growth on SBH the inducing compounds were more quickly removed from the cultures than on CS.

**Fig. 4:**
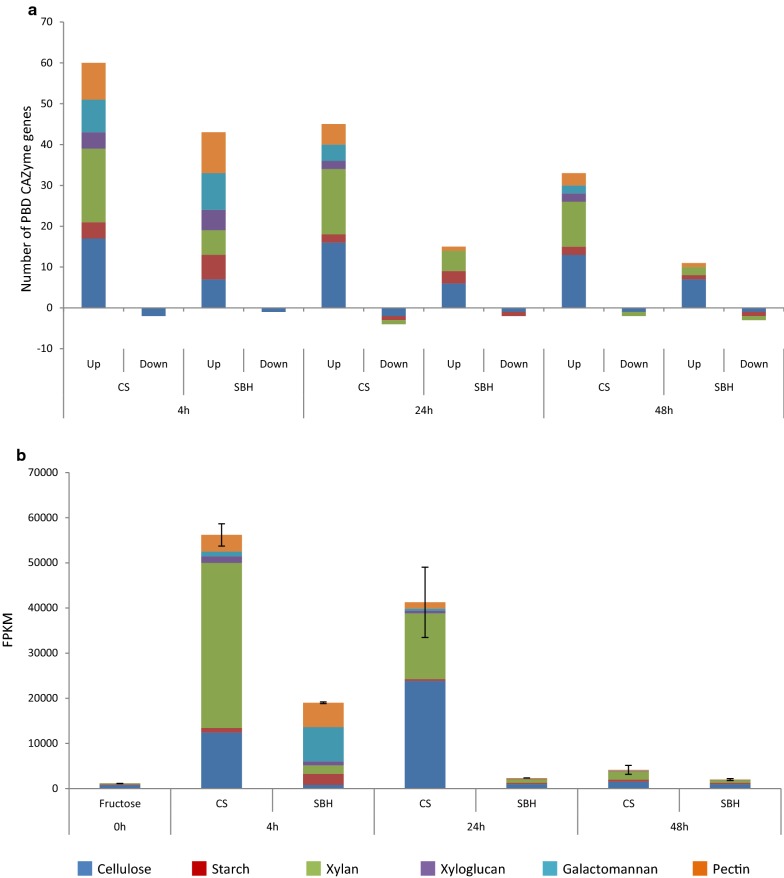
Comparison of PBD CAZyme gene expression in the reference strain (QM9414) on corn stover (CS) and soybean hulls (SBH). **a** Fold change analysis. Differentially expressed genes were those with a *p* value ≤ 0.05, fold change > 2.5 (log2foldchange > 1.32) compared to pre-culture and FPKM ≥ 18 in at least one condition. **b** Total expression analysis were performed with average FPKM level between three replicates in plant biomass and two replicates in pre-culture. Error bars represent the standard error on the total PBD CAZyme expression

Deep transcriptomic analysis (Fig. 4), in terms of which PBDs were induced and the level of total expression, helped to elucidate the patterns found in the PBD CAZymes cluster analysis (Fig. 3). CS induced more as well as higher total expression of cellulolytic and xylanolytic genes (Fig. 4a, b). In contrast, SBH induced more as well as higher total expression of mannanolytic, amylolytic and pectinolytic genes at the initial stage (4 h) (Fig. 4). This indicates that *T. reesei* is able to sense the major inducers from each substrate, adapting the expression of PBD CAZyme encoding genes to the substrate composition.

CS induced more PBD CAZyme encoding genes compared to SBH (Fig. 3) and to a higher level of total expression at all three time points (Fig. 4). The peak of PBD CAZyme gene expression on CS was at 4 h where the total expression of xylanolytic genes was highest (such as *xyn1/2/3/4*, *bxl1*, *aes1, agu1* and *abf2)* (Fig. 4b, Additional file 4), while cellulolytic gene expression was highest at 24 h (e.g., *cbh1/2*, *egl1/2/3//5* and two LPMOs *egl4* and *cel61b*) (Fig. 4b, Additional file 4). In general, in both substrates, the number of induced PBD CAZyme encoding genes and the total expression level decreased over time (Fig. 4), with a steeper decline over time in the total expression on SBH compared to CS (Fig. 4). The decrease over time led to a total level of PBD CAZyme gene expression at 48 h on both substrates that is comparable to the level in the d-fructose pre-cultures (Figs. 3, 4b) indicating that at 48 h little or no inducers were present in the cultures from both substrates. Considering that studies in *A. niger* [23, 24] and *T. reesei* [25] indicated that inducer concentrations below 1 mM already activate the regulatory systems, this suggests that in fact no inducers are present anymore.

**Fig. 5:**
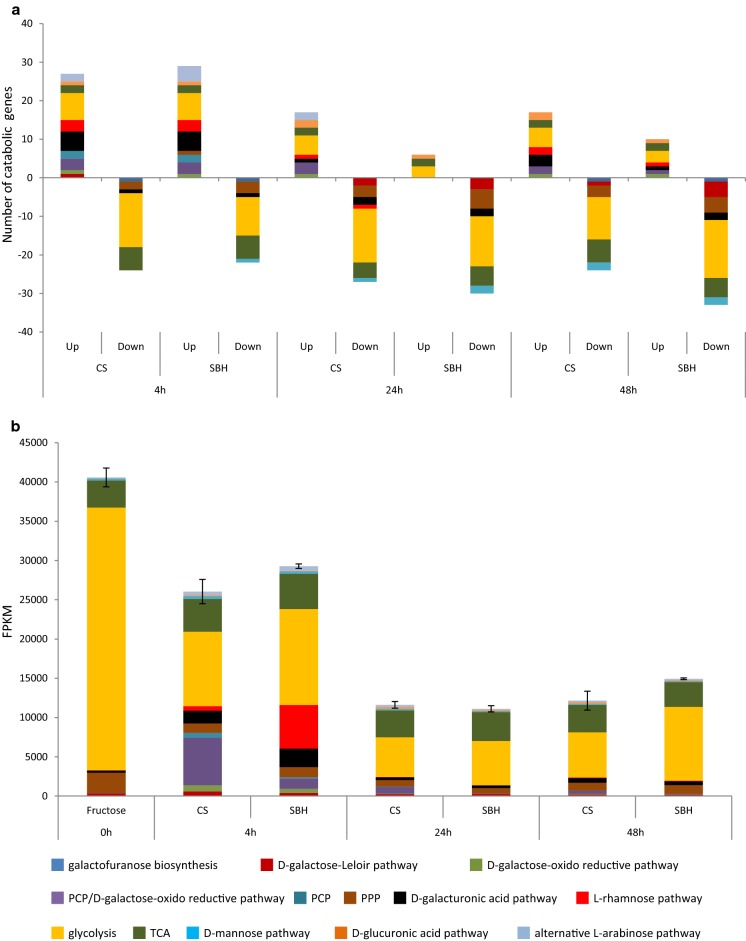
Comparison of reference carbon catabolic gene expression in corn stover and soybean hulls. **a** Fold change analysis. Differentially expressed genes were those with a *p* value ≤ 0.05, fold change > 2.5 (log2foldchange > 1.32) compared to pre-culture and FPKM ≥ 18 in at least one condition. **b** Total expression analysis were performed with average FPKM level between 3 replicates in plant biomass and 2 replicates in pre-culture. Error bars represent the standard error on the total C-catabolism expression

**Fig. 6:**
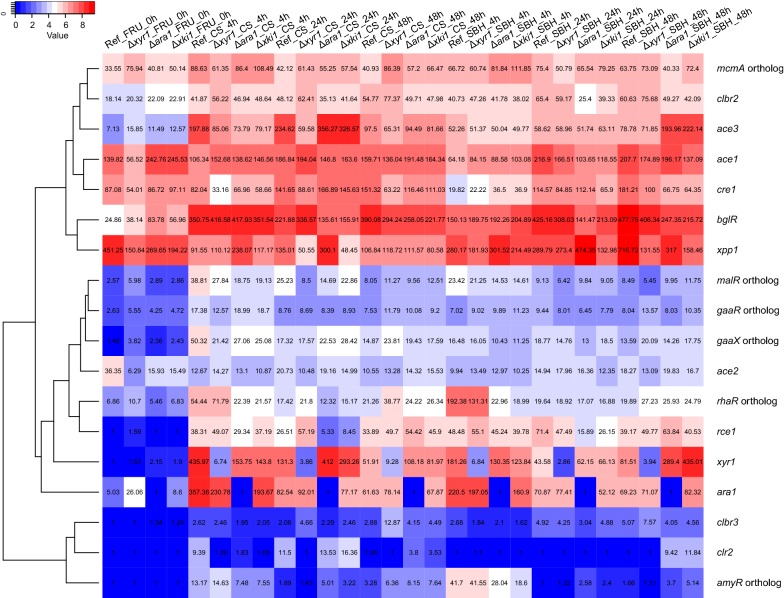
Hierarchical clustering (Euclidean distance) of regulator gene expression in all strains in d-fructose (pre-culture), corn stover and soybean hulls

Total expression of carbon catabolic genes in CS and SBH followed the PBD cellulolytic gene expression matches with CAZyme patterns where the expression decreased over time in both substrates, but for this gene group the total expression was comparable in both substrates at the same time point (Fig. 5). Similar to what was observed for the CAZyme genes, a more clear adaptation to substrate composition was observed at 4 h than at the later time points (Fig. 5). The total expression of genes involved in both the PCP and d-galactose oxido-reductive pathway (*xyl1*, *lad1* and *xdh1*) was higher in CS, while in SBH the total expression of the l-rhamnose pathway was higher (Fig. 5b). Transcriptional regulator encoding genes appeared to be similarly induced in both substrates (Fig. 6), but small differences were observed that also correlated with the composition of the substrates. CS induced more highly *xyr1* and *ace3* (both (hemi-)cellulolytic activators [26, 27]), while SBH higher expressed *rhr1* (ortholog of the *A. niger* rhamnose responsive regulator *rhaR* [28]) after 4 h, as well as *xpp1* (xylanase repressor [29]). The two substrates demonstrated clear differences in their induction pattern and physiological response and this led us to initially analyze the effects of the three deletion mutants by each substrate separately and then compare these analyses.

### XYR1 is the major TF regulating the degradation of corn stover, while ARA1 more strongly affects soybean hulls utilization

We initially analyzed the secretome profiles of the reference strain and what we considered phenotypically the most extreme mutant, Δ*xyr1*, by SDS-PAGE gel analysis on later time points (24 h and 48 h), where we expected to see clear patterns. *T. reesei* reference strain secreted a different protein pattern in each substrate, where CS showed more intense bands especially around 25 kDa. According to our expectations, *xyr1* deletion caused a severe secretome reduction in terms of bands intensity and range, in both substrates (Additional file 6).

**Fig. 7:**
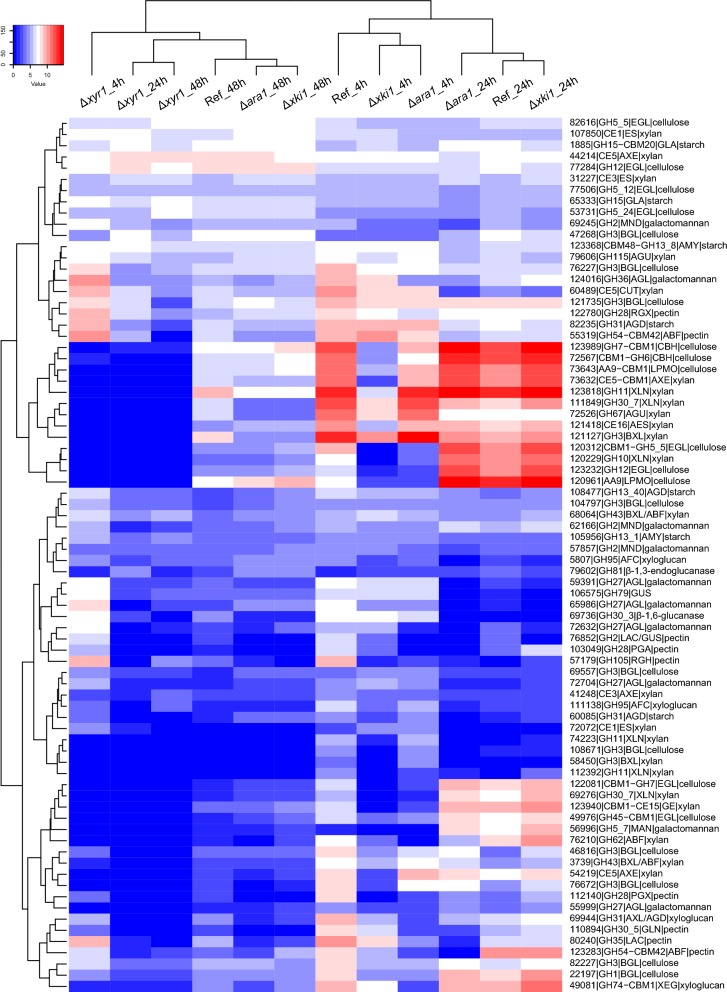
Hierarchical clustering (Euclidean distance) of PBD CAZyme gene expression in the *T. reesei* reference strain (QM9414) and deletion mutants Δ*xyr1*, Δ*ara1* and Δ*xki1* on corn stover (CS)

Clustering analysis showed that in CS, the 4 h and 24 h samples of the reference, Δ*ara1* and Δ*xki1* cluster as two related, time-dependent clusters, indicating that overall these strains behave very similar during growth on CS (Fig. 7). At 48 h, these strains still cluster together, but are now related to the 24 h and 48 h samples of Δ*xyr1*, characterized by an overall reduction in gene expression compared to the reference strain at the earlier time points (Fig. 7). The 4 h sample of Δ*xyr1* is clearly distinct from the other samples, with also an overall low expression of the CAZyme genes, but with some genes that are still higher expressed than at later time points in this strain. The results, therefore, support a central role for XYR1 in degradation of CS.

**Fig. 8:**
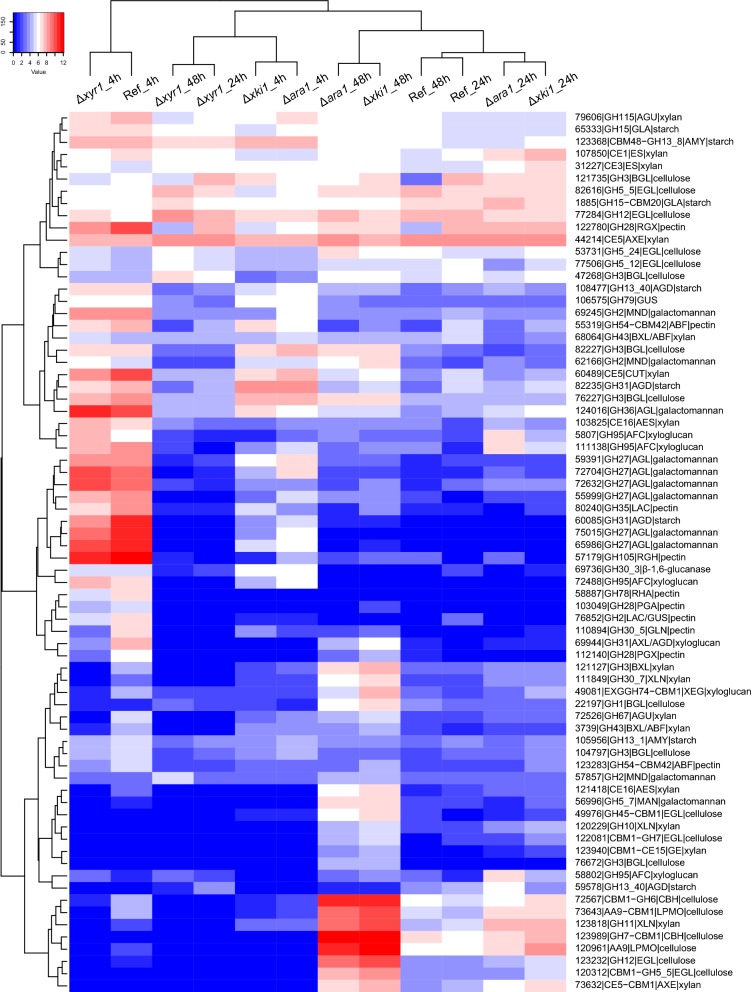
Hierarchical clustering (Euclidean distance) of PBD CAZyme gene expression in the *T. reesei* reference strain (QM9414) and deletion mutants Δ*xyr1*, Δ*ara1* and Δ*xki1* on soybean hulls (SBH)

A very different pattern was observed on SBH. Initially, there appears to be only a minor effect of *xyr1* as at 4 h, the Δ*xyr1* sample clusters with the 4 h reference strain sample (Fig. 8). However, at later time points the Δ*xyr1* samples are again characterized by overall very low gene expression compared to the reference strain at the earlier time point. The 4 h samples of Δ*ara1* and Δ*xki1* cluster together, but distant from the reference strain and Δ*xyr1* at this time point. However, they have some similarity to the later time points of Δ*xyr1*, characterized by a low expression of part of the CAZyme genes (Fig. 8). Some genes that are lowly expressed in the Δ*ara1* and Δ*xki1* strain at 4 h increase strongly in expression at the later time points, especially at 48 h, but this is not (Δ*xyr1*) or much less (reference strain) the case for the other strains. Overall, ARA1 appears to have a stronger role in SBH degradation, especially at the early time points. Interestingly, the metabolic mutant always clustered with Δ*ara1* at each time point on both substrates (Figs. 7, 8), which was unexpected, as the Δ*xki1* was expected to result in inducer accumulation and, therefore, upregulation of the ARA1 and XYR1 target genes, while Δ*ara1* was expected to reduce expression of a subset of those genes.

**Fig. 9:**
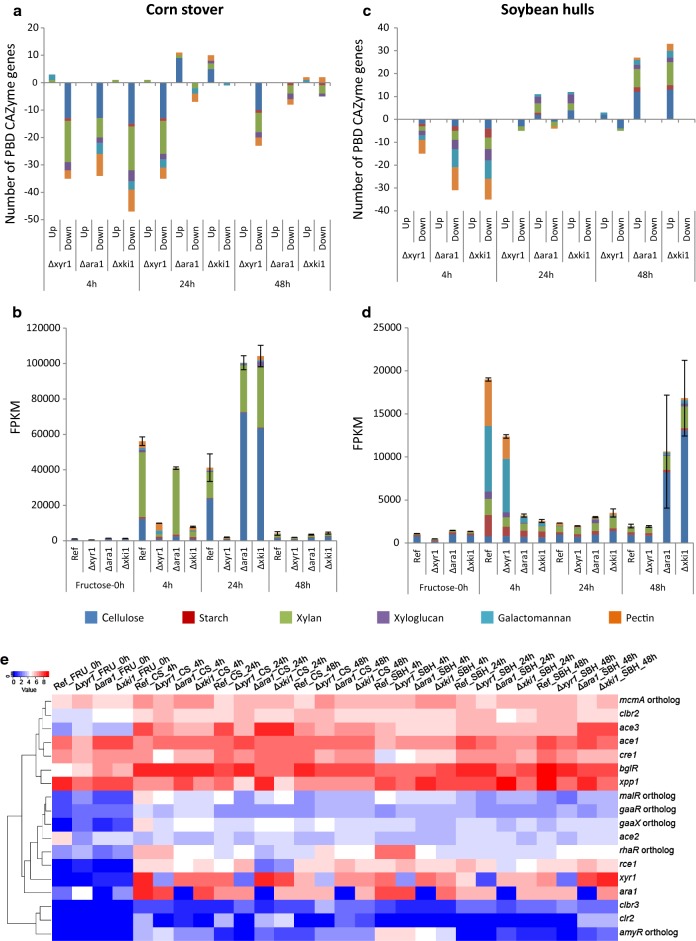
PBD CAZyme gene expression and TFs hierarchical clustering in the deletion mutants Δ*xyr1*, Δ*ara1* and Δ*xki1* compared to the reference strain in corn stover and soybean hulls. **a**, **c** Fold change analysis. Differentially expressed genes were those with a *p* value ≤ 0.05, fold change > 2.5 (log2foldchange > 1.32) compared to pre-culture and FPKM ≥ 18 in at least one condition. **b**, **d** Total expression analysis were performed with average FPKM level between 3 replicates in plant biomass and 2 replicates in pre-culture. Error bars represent the standard error on the total PBD CAZyme expression. E = TFs hierarchical clustering

More detailed analysis confirmed that Δ*xyr1* was the regulatory mutant more affected in CS, resulting in a lower total expression and number of PBD CAZymes in every time point (Fig. 9a, b). In contrast to CS, on SBH the *xyr1* deletion had a smaller effect on the response in all of the time points compared to the other mutants, in terms of number of PBD CAZymes induced and their total expression level (Fig. 9c, d). In SBH the biggest reduction in gene expression for Δ*xyr1* was observed at 4 h, but only approximately half of the PBD CAZyme genes were affected compared to CS and the total expression level was similar to the reference (Fig. 9). The *ara1* deletion had a much smaller effect on gene expression on CS, confirming the higher importance of XYR1 in CS utilization (Fig. 9a, b).

**Fig. 10:**
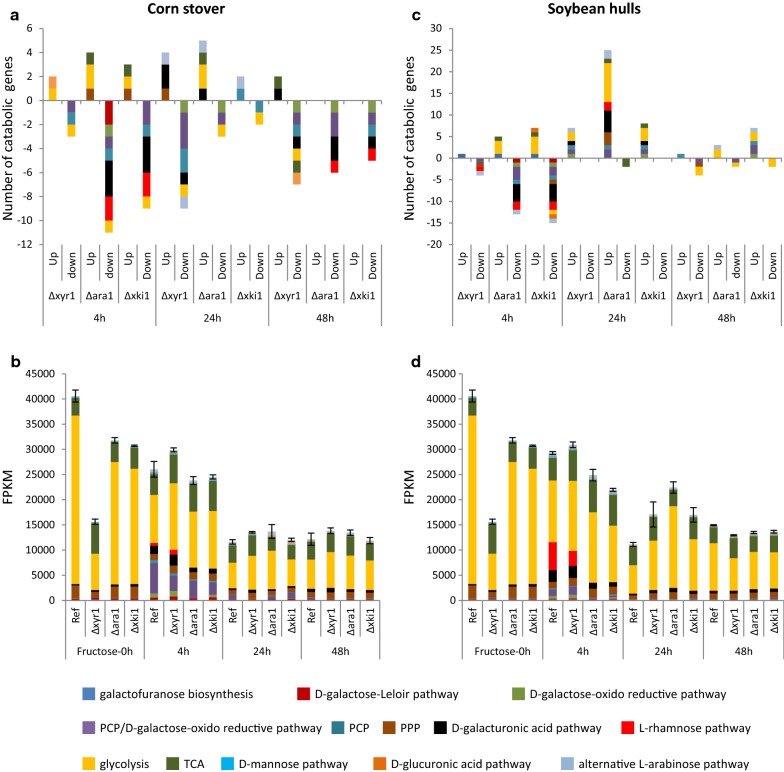
Carbon catabolic gene expression in the deletion mutants Δ*xyr1*, Δ*ara1* and Δ*xki1* compared to the reference strain in corn stover and soybean hulls. **a**, **c** Fold change analysis. Differentially expressed genes were those with a p value ≤ 0.05, fold change > 2.5 (log2foldchange > 1.32) compared to pre-culture and FPKM ≥ 18 in at least one condition.  **b**, **d**  Total expression analysis were performed with average FPKM level between 3 replicates in plant biomass and 2 replicates in pre-culture. Error bars represent the standard error on the total C-catabolism expression

The number of PBD CAZymes affected in both of regulatory mutants after 4 h on CS was similar but the total expression level in Δ*ara1* was still comparable to the reference (Fig. 9a, b). This was mainly due to xylanolytic genes, which remained expressed in Δ*ara1*, but were severely affected in Δ*xyr1*. At 4 h in CS, carbon catabolism was also affected by both mutations. The PCP genes were lowly expressed in both regulatory mutants, but the d-galactose Leloir and oxido-reductive pathways were affected more by the *ara1* deletion, while l-rhamnose and d-galacturonic pathways were affected only by the *ara1* deletion (Fig. 10a, b).

Similar to CS, the initial response on SBH was also affected by both regulatory mutants, but the effect was larger for Δ*ara1* than for Δ*xyr1* (Fig. 9c, d). In Δ*ara1* induction of mannanolytic and pectinolytic genes were particularly reduced (Fig. 9c, d). In addition, genes of the PCP, both d-galactose pathways and the d-galacturonic acid pathway (only at 4 h) were severely reduced in Δ*ara1*, while the l-rhamnose pathway genes were not or lowly expressed in Δ*ara1* (Fig. 10c, d). These pathways were not substantially affected in the *xyr1* mutant on SBH. This indicates that Δ*ara1* was not/less able to catabolize or release d-galactose, l-arabinose and l-rhamnose, confirming that ARA1 has a larger effect on SBH utilization than XYR1.

Surprisingly, in contrast to Δ*xyr1* and the initial response, at 24 h in CS and 24 h and 48 h in SBH Δ*ara1* induced more PBD CAZymes and to a higher level compared to the reference (Fig. 9). The same phenomenon was observed also for the Δ*xki1* strain and will be described in the following section.

### Deletion of *xki1* or *ara1* leads to higher expression of cellulolytic and xylanolytic genes in soybean hulls and corn stover

Clustering analysis (Figs. 7, 8) showed that Δ*xki1* and Δ*ara1* samples always clustered together and by time point in both substrates, suggesting a similar response from each of the mutants, which was confirmed by transcriptomic analysis where PBD CAZyme genes (Fig. 9) and carbon catabolic genes (Fig. 10) were similarly expressed. In Δ*xki1* and Δ*ara1*, the initial responses (4 h) to both substrates was severely affected, while at later time points (24 h in CS, 24 h and 48 h in SBH) more PBD CAZyme genes were induced and at higher level compared to the reference (Fig. 9). Remarkably, Δ*xki1* appeared to be more extreme in its responses to both substrates compared to Δ*ara1* (Fig. 9). The Δ*xki1* initial reduction in gene expression (4 h) to both substrates, but especially on CS, was more affected than that of Δ*ara1*, while during the PBD CAZyme gene expression peaks (24 h in CS, 24 h and 48 h in SBH) more PBD CAZyme genes were highly expressed compared to Δ*ara1* (Fig. 9a, c). At these time points, and especially in SBH, genes of the PCP, d-galactose pathways and d-galacturonic acid pathway were also higher expressed in both strains (Fig. 10b, d). Many of the genes that were upregulated in one or both of the strains (Δ*xki1* and/or Δ*ara1*) and substrates (CS and/or SBH) are mainly regulated by XYR1 (Table 1).

**Table 1:** Support for *xyr1*-activation of the PBD CAZymes that were higher expressed in the Δ*xki1* mutant at later time-points in corn stover (CS_24 h) and soybean hulls (SBH_48 h)

Trire2_geneID	Gene name	Activity	Regulated by	CS_24 h	SBH_48 h
3739		BXL/ABF	XYR1	Yes	Yes
22197	*cel1b/bgl1b*	BGL	XYR1	Yes	Yes
46816	*cel3d/bgl3d*	BGL	Unknown	Yes	No
49081	*cel74a*	XEG	ARA1/XYR1	Yes	Yes
49976	*egl5/cel45a*	EGL	XYR1	Yes	Yes
56996	*man1*	MAN	XYR1	No	Yes
60489		CUT	Unknown	No	Yes
62166		MND	Unknown	No	Yes
69944		AXL/AGD	ARA1 (XYR1)	No	Yes
72526	*glr1/agu1*	AGU	XYR1	No	Yes
72567	*cbh2/cel6a*	CBH	XYR1		Yes
73632	*axe1*	AXE	XYR1	No	Yes
73643	*egl4/cel61a*	LPMO	XYR1	Yes	Yes
76210	*abf2*	ABF	ARA1(XYR1)	Yes	No
76227	*cel3e/bgl3e*	BGL	Unknown	No	Yes
76672	*bgl1/cel3a/bgl3a*	BGL	XYR1	No	Yes
82227	*cel3c/bgl3c*	BGL	XYR1	No	Yes
82235		AGD	XYR1	No	Yes
103049		PGA	ARA1(XYR1)	Yes	No
108477		AGD	Unknown	No	Yes
111849	*xyn4*	XLN	XYR1	No	Yes
112140	*pgx1*	PGX	Unknown	No	Yes
120229	*xyn3/xyn10a*	XLN	XYR1	No	Yes
120312	*egl2/cel5a*	EGL	XYR1	No	Yes
120961	*cel61b*	LPMO	XYR1	No	Yes
121127	*bxl1/xyl3a*	BXL	XYR1	No	Yes
121418	*aes1*	AES	XYR1	No	Yes
121735	*cel3b/bgl3b*	BGL	XYR1	No	Yes
122081	*egl1/cel7b*	EGL	XYR1	No	Yes
122780	*rgx1*	RGX	ARA1	No	Yes
123232	*egl3/cel12a*	EGL	XYR1	No	Yes
123283	*abf1*	ABF	ARA1(XYR1)	No	Yes
123818	*xyn2/xyn11a*	XLN	XYR1	No	Yes
123940	*cip2*	GE	XYR1	No	Yes
123989	*cbh1/cel7a*	CBH	XYR1	Yes	Yes
124016	*agl2*	AGL	ARA1	No	Yes

DE genes were assigned to be regulated by ARA1 and/or XYR1 according to literature [9, 22, 27, 30] or this study

ARA1/XYR1, both ARA1 and XYR1 have similar impact in regulation; ARA1 (XYR1), ARA1 has a bigger impact in regulation compared to XYR1

In CS at 24 h (where the PBD CAZyme gene expression peaked) in both strains, mainly cellulolytic and xylanolytic genes, but also a few pectinolytic genes were more induced, while additionally in Δ*xki1* a few xyloglucanolytic genes were higher expressed (Fig. 9a, b). Many of these genes are described as XYR1 target genes [9, 22, 27, 30], including the major cellulolytic genes (e.g., *bgl1, egl1/2/3/4/5* and *cbh1/2*), xylanolytic genes (e.g., *xyn3/4, axe1* and the candidate β-xylosidase/α-l-arabinofuranosidase *Trire2_3739),* one pectinolytic gene (*pgx1*) and one mannanolytic gene (*man1*) (Table 1). Only in the Δ*xki1* strain, two arabinanolytic (e.g., *abf2*) gene and one PCP gene (*lxr3*) were higher expressed, which were also reported to be XYR1 regulated [9, 22, 30] (Table 1). Expression of *xyr1* was also higher, while the cellulase repressor *rce1* [31] was lower expressed (Fig. 6).

In SBH at 48 h, Δ*xki1* and Δ*ara1* had their highest PBD CAZyme gene expression, which included pectinolytic, amylolytic, cellulolytic and xylanolytic genes (Fig. 9). Similar to CS, *xyr1* and *ace3* were also higher expressed in Δ*xki1* and Δ*ara1* (Fig. 6), and many of the genes upregulated in SBH at 48 h in Δ*xki1* and Δ*ara1* have been described as XYR1 target genes [9, 22, 27, 30] (Table 1). These included cellulolytic (e.g., *egl1/2/3/4/5* and *cbh1/2*), xylanolytic (e.g., *xyn3/45, bxl1, axe1* and *aes1),* and mannanolytic (e.g., *man1* and the candidate β-mannosidase *Trire*-*2_62166*) genes [8, 9, 18, 22, 27, 30] (Table 1). In addition to these, *abf1*, the candidate β-xylosidase/α-l-arabinofuranosidase *Trire2_3739, bgl1* (Table 1) and two PCP genes, *xyl1* (XYR1 regulated) and *lxr3* were higher expressed in Δ*xki1*, but not Δ*ara1* [9].

## Discussion

In this study we evaluated the transcriptomic response of the *T. reesei* reference strain, two regulatory mutants and a metabolic mutant during growth on two plant biomass substrates, corn stover (CS, monocot) and soybean hulls (SBH, dicot). Our data showed that substrate composition strongly affected PBD CAZyme expression, resulting in higher induction of a broader set of CAZyme encoding genes in CS compared to SBH (Figs. 3,  4). This higher (hemi-)cellulolytic gene expression matches with the substrate composition (CS is richer in hemicellulose, especially arabinoxylan) (Additional file 1), as well as the more complete set of CAZymes in the *T. reesei* genome for CS degradation [6]. In addition, PBD CAZyme and carbon catabolism related gene expression decreased over time earlier in SBH (Figs. 4,  5), suggesting that release of inducing compounds by *T. reesei* finished earlier on SBH. Indeed, *T. reesei* has only a few enzyme activities [6] for efficient degradation of SBH, such as low numbers of pectinases and α-xylosidases and a complete lack of endo-arabinanases and feruloyl esterases. This may have resulted in fewer inducers released from SBH and consequently in lower induction of a narrower set of PBD CAZyme encoding genes. d-xylose, which has been demonstrated to be a major inducer of the (hemi-)cellulolytic system in *T. reesei* [25], is mainly α-linked in xyloglucan in SBH, whereas it is β-linked in xylan in CS [32]. In CS, the major β-xylosidase *bxl1* was highly expressed at all three time points, while no β-xylosidase expression was found at any time point on SBH (Additional file 4). *T. reesei* has one putative α-xylosidase [6], which was expressed after 4 h (but not 24 h and 48 h) on SBH, possibly limiting the release of the α-linked d-xylose from SBH more so than the β-linked d-xylose in CS (Additional file 4). This was supported by a study, in which the addition of extra α-xylosidase activity to a *T. reesei* commercial enzyme cocktail improved significantly the amount of d-xylose released from pea or tamarind xyloglucan [33].

The data of this study provides leads to improve the production of a commercial *T. reesei* enzyme cocktail, such as by the choice of substrate on which the enzymes are produced and the time after which enzymes are harvest. CS appears a better substrate to obtain a (hemi-)cellulolytic cocktail, while SBH is better for a pectinolytic, amylolytic and mannanolytic cocktail (Fig. 4). In both substrates, the overall expression level was higher at the earliest time point (4 h), suggesting that high enzyme production will not be sustained over time unless the consumption of potential inducers can be impaired such as in the Δ*xki1* mutant. A similar pattern was observed in *Podospora anserina* using comparable methodology including the same substrates [34], where CS induced predominantly (hemi-)cellulases, while SBH induced more amylolytic and pectinolytic genes. However, in *P. anserina,* SBH was the broader and higher PBD CAZyme gene inducing substrate compared to CS. This indicates that the inducing effect of crude substrates can be species-specific, depending on the genome content and plant biomass degradation strategy. Nevertheless, commonalities can be found in the responses of fungi from diverse biotypes. Analysis of the *T. reesei* PBD CAZyme genes expressed in both substrates resulted in a core set of 35 shared enzyme activities (data not shown). Of these activities, 14 were in common with the 18 activities in the core set of *P. anserina* and saprobic basidiomycetes [34, 35]. These 14 activities included three cellulolytic (LPMO, CBH and EGL), one amylolytic (AGD), four xylanolytic (AXE, XLN, ABF, BXL), three mannanolytic (MAN, MND and LAC), two pectinolytic (LAC and ABF) and one xyloglucanolytic (XEG) gene. This core enzyme set could be considered as a general response of fungi from diverse biotypes to commonly found components of plant biomass.

We showed that particularly (hemi-)cellulolytic genes were more induced in CS, most likely due to the higher expression of the two (hemi-)cellulolytic regulators *xyr1* and *ace3* (Fig. 6). However, a higher expression of a TF does not necessarily lead to higher expression of its target genes, because they can be regulated also at post-transcriptional level, as has previously been shown for XYR1 and other TFs [8]. However, our data demonstrates low expression for *clr2* in all conditions, suggesting a different function in *T. reesei* for CLR2 compared to *Neurospora crassa* and several Aspergilli, where it has been described as a cellulolytic and mannanolytic regulator [36–38]. Overexpression of *clr2* in *T. reesei* did not result in substantial enhancement in cellulase and xylanase activity [26]. The orthologs of the *A. niger* d-galacturonic acid regulators *gaaR* and *gaaX* [39] were also poorly expressed in most of the conditions (Fig. 6), suggesting that the mechanism with which *T. reesei* responds to d-galacturonic acid may differ from *Botrytis cinerea* and *A. niger* [6, 8].

Our data showed that all mutations, both regulatory and catabolic, severely affected the initial response (4 h) to both substrates (Figs. 9, 10). This suggests an initial delay in the release and utilization of enough easily metabolized sugars/inducers at 4 h by the mutants, resulting in a lack of energy, lower co-factor regeneration (especially for Δ*xki1*), such as NADH and NADPH that are necessary for the activity of many oxidoreductases of catabolic pathways, and carbon to synthesize the necessary proteins to degrade plant biomass. Lower PBD CAZyme gene expression was also observed in a study performed with comparable methodology in *A. niger,* where Δ*xlnR* and Δ*xkiA* mutants severely affected PBD expression in both CS and SBH at the early stage [40]. Comparison with this *A. niger* study is particularly informative as similar conditions (with respect to time-points and substrates) and mutants were used.

Our study demonstrates that *xyr1* is the major TF affecting CS utilization, where its deletion caused a massive reduction of PBD CAZyme gene expression at 4 h and 24 h, especially of cellulolytic and xylanolytic genes (Fig. 9). This matches with the substrate composition (Additional file 1) and the function described for this regulator, as indeed CS is richer in (hemi-)cellulose and XYR1 is the main (hemi-)cellulolytic activator [9, 22, 27]. This confirms a previous study, in which another (hemi-)cellulose-rich substrate (wheat bran) and a *T. reesei* Rut-C30 Δ*xyr1* mutant were used [18].

In contrast, at 4 h in SBH, substrate utilization was more affected by the *ara1* deletion resulting in a more severe reduction of PBD CAZyme expression. This dependence of the early response to SBH on ARA1 is most likely due to the fact that ARA1 responds to l-arabinose and d-galactose [9] (inducers which SBH is richer in Additional file 1). During the later response (24 h and 48 h), other TFs indirectly compensated for this loss, such as the higher expression of the (hemi-)cellulolytic activators *xyr1* and *ace3* [8, 22, 26], or the lower expression of the cellulase repressor *rce1* [31] (Fig. 6). Supporting this role of these regulators, cellulolytic and xylanolytic genes were higher expressed in Δ*ara1* at the later time points (Fig. 9).

Our study is the first which highlights the importance of TFs other than XYR1, in a pectin- and mannan rich substrate such as SBH for *T. reesei*. Most of the previous studies where *xyr1* was deleted used substrates that did not contain pectin or mannan, such as lactose, cellulose or xylan-rich crude plant biomass [15, 18, 22, 27, 30, 41–44]. The use of SBH in our study not only showed that XYR1 was not the major regulator on this substrate, but also that XYR1 partially regulates a few pectinolytic and mannanolytic genes (Additional file 4). This suggests that the function of XYR1 is not limited to cellulolytic and xylanolytic genes [27], but is broader than what has so far been described [8, 9, 27, 30]. Another broader role for XYR1 was reported by Ma et al. [18], where XYR1 appeared to regulate not only (hemi-)cellulolytic genes, but also genes encoding non-enzymatic cellulose active enzymes, sugar transporters and heat shock proteins.

In *A. niger*, XlnR (*xyr1* ortholog) and AraR (functional homolog of *ara1*) can compensate for each other’s loss, by inducing the target genes of the deleted regulator, most likely due to a similar binding motif [45]. In contrast, *T. reesei* XYR1 and ARA1 are not able to compensate for each other loss, probably because they are not closely related and, therefore, also bind to distinct promoter sequences [8, 9]. XYR1 appeared to express its own target genes, mainly (hemi-)cellulolytic genes [9, 22, 27], to a higher level in the Δ*ara1* strain, but not ARA1-target genes (Fig. 9).

The residual growth of Δ*xki1* on l-arabinose (which should result in a block of the PCP, Fig. 2), suggests the presence of another catabolic pathway in *T. reesei* to partially catabolize l-arabinose instead of the PCP. In contrast, the *A. niger* xylulokinase mutant cannot grow on l-arabinose [12], indicating a difference in the organization of these pathways between these two fungi. Blast analysis revealed that the *T. reesei* genome contains orthologs for the non-phosphorylative l-arabinose pathway from the bacterium *Azospirillum brasiliense* [46] (referred to as “alternative l-arabinose pathway” in our analysis). Whether this putative alternative l-arabinose pathway is responsible for the further catabolism of l-arabinose in Δ*xki1* strain (Fig. 10) requires additional studies.

In Δ*xki1*, the putative block of the PCP resulted in higher PBD CAZyme expression at later time points on both substrates (24 h in CS and 48 h in SBH) (Fig. 9). Many of these genes were described as XYR1 regulated [9, 27, 30] (Table 1) and were also upregulated in Δ*ara1*. A similar inducing effect was also observed in *A. niger* Δ*xkiA* [40], where accumulation of inducers [12], such as xylitol and l-arabitol, resulted in more PBD CAZyme genes that were highly expressed compared to the reference strain at later time points. However, this was limited to genes acting on pectin or with an activity that could be involved in the degradation of several substrates. This inducer(s) accumulation could also be the explanation for our results with *T. reesei,* which resulted in higher expression of the (hemi-)cellulolytic regulator *xyr1* (Fig. 6) and its target genes [9, 27, 30]. Xylitol and l-arabitol accumulation was already reported for other *T. reesei* PCP-knockout strains (Δ*xyl1*, Δ*lad1*, Δ*lxr3*) [13]. This inducer(s) accumulation (such as by xylitol and l-arabitol) is a possible explanation for the higher expression of PBD CAZymes observed also in the Δ*ara1* strain. However, in this case, where a TF is missing, we cannot exclude the involvement of other regulatory mechanism(s) such as secondary/backup regulatory system(s). It was previously shown in *A. niger* that XlnR and AraR have an antagonistic effect on each other and that deletion of one, increases expression of the target genes of the other [47, 48]. Considering that growth on solid media, where sugars other than pentoses were available, was comparable to the reference strain (Fig. 2), deletion of *xki1* could be used to improve production of *T. reesei* enzyme cocktails at industrial scale by limiting the catabolism of pentose inducers from a crude plant biomass substrate, potentially sustaining the induction of the enzyme encoding genes longer.

## Conclusion

CS induces a broader and higher expression of PBD CAZyme encoding genes in *T. reesei*, while SBH could be used to induce an enzyme cocktail that is richer in pectinolytic and mannanolytic enzymes. XYR1 is the major TF affecting CS utilization, while ARA1 affects more SBH utilization. Blocking the PCP by deleting *xki1* leads to higher expression of PBD CAZymes at later time points in the cultures, which could lead to a novel strategy to improve the enzyme cocktail production at industrial level.

## Materials and methods

### Strains, media, and growth conditions

*Trichoderma reesei* QM9414 (ATCC 26921) [49] was used as reference strain and compared to CBS 143327 (Δ*xyr1*) [22], CBS143330 (Δ*ara1*) [9] and CBS143332 (Δ*xki11*) (this study) in all experiments. All *T. reesei* plate cultures were incubated at 28 °C on PDA (Difco) for sporulation, or minimal medium (MM) [50] with 18 g/L Select agar (Invitrogen) during the transformation or growth profiling (in this case Na-citrate was removed). The growth profile was performed on MM with 25 mM d-glucose (Sigma), d-fructose (Sigma), d-xylose (Sigma), l-arabinose (Sigma), xylitol (Sigma), l-arabitol (Sigma), d-galactose (Sigma), lactose (Sigma), 1% arabinan (Megazyme), wheat arabinoxylan (Megazyme), apple pectin (Sigma), avicel (Fluka), 3% soybean hulls and corn stover in 9 cm Petri dishes. Duplicate plates were inoculated with 2 μL containing 1 × 10^3^ spores, which were pre-germinated overnight in MM with 1% d-fructose and 0.1% peptone, and incubated in the dark for at least 5 days at 28 °C. Pre-germination facilitates replicable growth on C-sources where *T. reesei* spores germinate infrequently or do not germinate. Independent deletion strains were generated and tested for growth on a subset of C-sources to confirm the reliability of attributing the observed phenotypes to deleted gene (data not shown). We selected one strain to use in further studies and deposited these at the Westerdijk Fungal Biodiversity Institute collection, with strain number as indicated above.

A transfer experiment was performed for transcriptomics. 250 mL of complete medium (CM) [51] containing 2% d-fructose in 1 L Erlenmeyer flasks was inoculated with 2.5 × 10^8^ fresh spores, harvested from a PDA plate, and incubated in a rotatory shaker at 28 °C for 20 h at 250 rpm. The mycelium was harvested by filtration, washed with liquid Mandels Andreotti medium (MA) [52] (without carbon source) and 2.5 g mycelium (wet weight) was transferred to 250 mL Erlenmeyer flasks containing 50 mL MA with 1% of soybean hulls or corn stover, and incubated in a rotatory shaker at 28 °C and 250 rpm. After pre-culturing and after 4 h, 24 h, and 48 h of incubation in CS or SBH, the mycelium was harvested by vacuum filtration, dried between tissue paper, directly frozen in liquid nitrogen and stored at − 45 °C [53]. All experiments were performed in triplicates, with the exception of pre-cultures, which were performed in duplicates.

### Molecular biology methods

The hygromycin B^R^ cassette was amplified from the plasmid pLH1hph [54] and fused with 1 kb flanking regions up- and downstream of the *xki1* gene by fusion-PCR and purified as described by Klaubauf et al. [53]. This *xki1* deletion cassette was used to transform spores of *T. reesei* QM9414 Δ*tku70* [55] by electroporation as described by Schuster et al. [56], using a Bio-Rad Gene Pulser Electroporator System set at 1.8 kV, 800 Ω and 25 μF. DNA from transformants was screened by PCR for the absence of *xki1* and the correct positioning of the insert as described by Klaubauf et al. [53]. The absence of ectopic integrations was confirmed by Southern blot (Additional file 2) using DIG Easy Hyb kit (Roche) and Anti-Digoxigenin-AP, Fab fragments (Roche) with a probe designed to hybridize to part of the hygromycin resistance gene sequence and amplified with the PCR DIG Probe Synthesis Kit (Roche), according to the manufacturer protocols. Primers used for PCR reactions are listed in Additional file 3.

Total RNA was extracted from mycelium ground in a Tissue Lyser (QIAGEN) using TRIzol reagent (Invitrogen) according to the manufacturer’s instructions. RNA integrity and quantity were analyzed on a 1% agarose electrophoresis gel and with the RNA6000 Nano Assay, using the Agilent 2100 Bioanalyzer (Agilent Technologies) [53].

Culture filtrate samples (10 mL) were taken after 24 h and 48 h and centrifuged for 10 min, at ~ 10,000×*g*, 4 °C to separate the solid fraction from the supernatant and stored at − 20 °C. 150 µL of these culture filtrates from reference and Δ*xyr1* strains at 24 h and 48 h of cultivation were added to 50 µL of loading buffer (10% of 1 M Tris–HCl, pH 6.8; 42% Glycerol, 4% (w/v) SDS; 0.02% (w/v) bromophenol blue; 4% of 14.7 M Mercaptoethanol), boiled for 2 min to denature the proteins, cooled on ice for 2 min and centrifuged at ~ 10,000×*g* for 2 min to remove insoluble material. 20 µL was then loaded onto 12% (w/v) acrylamide SDS-PAGE gels and a molecular weight marker (Bio-Rad unstained marker) was used to identify the molecular mass of the protein bands. The gels were silver stained [57] and documented using the HP scanner 4400c.

### RNA sequencing and read mapping

RNA samples (5–41 μg DNase-treated total RNA) were processed by Joint Genome Institute. RNA sequencing was performed using Illumina HiSeq 2500 (yield 1 TB of 1 × 101 bp). Raw fastq file reads were filtered and trimmed using the JGI QC pipeline. Using BBDuk [BBDuk: https://sourceforge.net/projects/bbmap/] raw reads were evaluated for artifact sequence by kmer matching (kmer = 25), allowing 1 mismatch and detected artifact was trimmed from the 3′ end of the reads. RNA spike-in reads, PhiX reads and reads containing any Ns were removed. Quality trimming was performed using the phred trimming method set at Q6. Reads under the length threshold were removed. Filtered reads from each library were aligned to the reference genome (https://genome.jgi.doe.gov/Trire2/Trire2.home.html) using HISAT version 0.1.4-beta [58]. featureCounts [59] was used to generate the raw gene counts using gff3 annotations. On average 94% of the reads mapped to the genome. The RNA-seq data have been deposited at the Sequence Read Archive at NCBI with individual sample BioProject Accession numbers (PRJNA440083 to PRJNA440152 and PRJNA442529 to PRJNA442538.

### RNA-seq data analysis

Raw gene counts were used to evaluate the level of correlation between biological replicates using Pearson’s correlation matrix (Additional file 5). DESeq 2 (version 1.10.0) [60] was used to determine which genes were differentially expressed (DE) between pairs of conditions. The parameters used to call a gene DE between conditions were adjusted *p* value ≤ 0.05, foldchange > 2.5 (log2foldchange > 1.32) and FPKM ≥ 18 in at least one condition. Genes with FPKM values < 18 in every condition were considered lowly expressed and ignored in the analysis.

Transcriptomics analysis focused only on genes encoding PBD CAZyme (plant biomass degrading enzymes), carbon catabolic enzymes and TFs (Additional file 4), using the list we built previously [9, 61].

PBD CAZyme (which had FPKM ≥ 18 in at least one of the conditions for a particular heatmap) or TF (all were clustered without regard to their minimum FPKM value) genes were hierarchically clustered using the heatmap.2 function (with default parameters: Euclidean distance, and complete linkage clustering method) from the gplots_3.0.1 package in R statistical language and environment 3.4.0. Log2 FPKM values were used for the color gradient of the heatmap and FPKM values < 1 were assigned to 1.

## Additional files

**Additional file 1.** Table with sugar composition of corn stover (CS) and soybean hulls (SBH). According to literature lignin content (W/W) is around 15–21% in CS [19] and 1–4% in SBH [20].

**Additional file 2.** Southern blot of Δ*xki1* strains. Positive gene deletion required bands of 6.4 kb and 10.8 kb for Δxki1. Both Δ*xki1* strains were correct.

**Additional file 3.** Table with PCR primers used in this study.

**Additional file 4.** Enzyme activity abbreviations and transcriptome dataset tables. Table with enzyme activity, their abbreviations and their predicted target substrate used in this study. The same color scheme for target substrates was used in the main text. A gene to be assigned as expressed more on a substrate (CS or SBH) requires a foldchange > 2.5, *p* ≤ 0.05 and at least 18 FPKM of expression in at least one condition. Genes with expression < 18 FPKM in each condition were excluded from subsequent analysis, because they were considered to be too poorly expressed.

**Additional file 5.** Pearson correlation matrix of *Trichoderma reesei* transcriptomes. Raw gene counts were used to evaluate the level of correlation between biological replicates using Pearson’s correlation. Pearson correlation matrix were performed in R (v3.4.0) statistical language and environment, the core function from the stats base package and the corrplot (v 0.77) package were used for the analysis. One sample (reference strain on SBH at 48 h) was removed from the dataset because it correlated poorly with its replicates.

**Additional file 6.** Analysis of the protein banding patterns from the three replicate culture supernatants of *T. reesei* reference (ref) and Δ*xyr1* strains cultured with either corn stover or soybean hulls for 24 h and 48 h. The same volume of culture supernatant was loaded for all samples.

## Abbreviations

TF: transcription factor
CAZy: carbohydrate-active enzymes database
CAZyme: carbohydrate-active enzyme
CCR: carbon catabolite repression
PBD: plant biomass degrading enzyme
CS: corn stover
SBH: soybean hulls
PCP: pentose catabolic pathway
PPP: pentose phosphate pathway
ABF: α-l-arabinofuranosidase
AES: acetyl esterase
AGD: α-1,4-glucosidase
AXE: acetyl xylan esterase
BXL: β-1,4-xylosidase
CBH: cellobiohydrolase
EGL: β-1,4-endoglucanase
LAC: β-1,4-galactosidase
LPMO: lytic polysaccharide monooxygenase
MAN: β-1,4-endomannanase
MND: β-1,4-mannosidase
XEG: xyloglucan-active endo-β-1,4-glucanase
