# Supplementary material for: Deletion of either the regulatory gene ara1 or metabolic gene xki1 in Trichoderma reesei leads to increased CAZyme gene expression on crude plant biomass
Source: Biotechnol Biofuels. 2019 Apr 9;12:81. doi: 10.1186/s13068-019-1422-y (PMC6454604; doi:10.1186/s13068-019-1422-y)
Supplement: Supplementary file 1 — Additional file 1. Table with sugar composition of corn stover (CS) and soybean hulls (SBH). According to literature lignin content (W/W) is around 15–21% in CS [19] and 1–4% in SBH [20]. [file 13068_2019_1422_MOESM1_ESM.pdf]

Additional file 1. Sugar composition of corn stover (CS) and soy bean hulls (SBH).
According to literature lignin content (W/W) is around 15-21% in CS [18] and 1-4% in SBH [19]

Mol %	L-rhamnose	D-fucose	L-arabinose	D-xylose	D-mannose	D-galactose	D-glucose	Uronic acid	main polysaccharides
**CS**	0.4	0	4.6	34.9	0.7	1.7	53.4	4.3	cellulose and arabinoxylan
**SBH**	1	0	8.4	15	7.1	4	50	15.9	cellulose, pectin, xyloglucan and mannan
