# Supplementary material for: Deletion of either the regulatory gene ara1 or metabolic gene xki1 in Trichoderma reesei leads to increased CAZyme gene expression on crude plant biomass
Source: Biotechnol Biofuels. 2019 Apr 9;12:81. doi: 10.1186/s13068-019-1422-y (PMC6454604; doi:10.1186/s13068-019-1422-y)
Supplement: Supplementary file 2 — Additional file 2. Southern blot of Δxki1 strains. Positive gene deletion required bands of 6.4 kb and 10.8 kb for Δxki1. Both Δxki1 strains were correct. [file 13068_2019_1422_MOESM2_ESM.pdf]

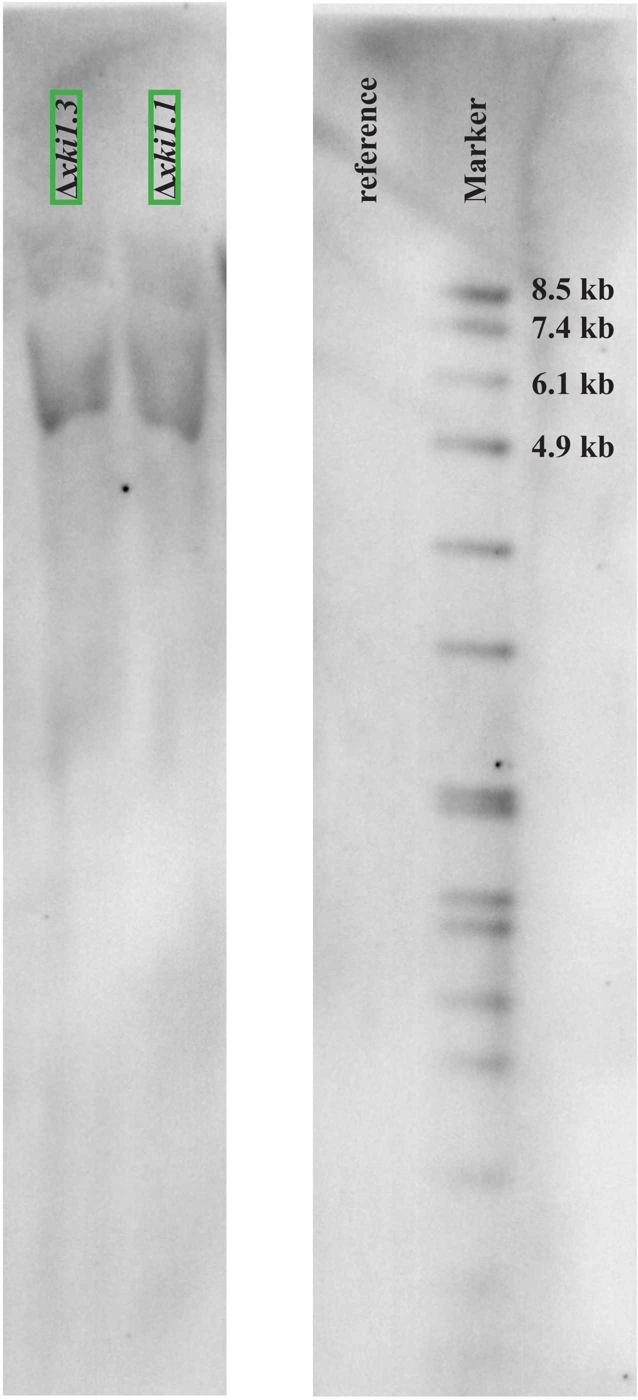

Additional file 2. Southern blot of Δ*xki1* strains.
Positive gene deletion required bands of 6.4kb and 10.8kb for Δ*xki1*. Both Δ*xki1* strains were correct.
