# Supplementary material for: Deletion of either the regulatory gene ara1 or metabolic gene xki1 in Trichoderma reesei leads to increased CAZyme gene expression on crude plant biomass
Source: Biotechnol Biofuels. 2019 Apr 9;12:81. doi: 10.1186/s13068-019-1422-y (PMC6454604; doi:10.1186/s13068-019-1422-y)
Supplement: Supplementary file 3 — Additional file 3. Table with PCR primers used in this study. [file 13068_2019_1422_MOESM3_ESM.pdf]

Additional file 3. PCR primers used in this study.

Primer	Sequence	PCR
Pa-hph-F	CTTGTCCCTTCCAGGCAAGC	Amplification of hph from plasmid
Pa-hph-R	CGGTGATGTCGGCGATATAGG	Amplification of hph from plasmid
xki1-5'F	gtaacgccagggttttcccagtcacgacgGACGCAGAGGTCAACAAGAGG	Amplification of 5' flank
xki1-5'R	ccatattgatgtaaggtagctctcggatccAAGCTGCTCTCAAAGATGCTTCG	Amplification of 5' flank
xki1-3'F	catctgtgctgcaaatccaagaaagtcgacAACCATGCACGTCGCTAATCC	Amplification of 3' flank
xki1-3'R	gcggataacaatttcacacaggaaacagcAGCCTAAAGTTGCCTGTTTTCGG	Amplification of 3' flank
xki1-5'F-nest	ACGGACACAAGCATTAGAGATGG	Fusion PCR
xki1-3'R-nest	AGCAGTTCTTCCCAACTTGCC	Fusion PCR
xki1-F	CAAGTCTACGCAAACGCAAAGC	Absence of *xki1*
xki1-R	AATGAGGTCGTCAAACGTCTCG	Absence of *xki1*
xki1-5'F-trans	GACGCAGAGGTCAACAAGAGG	Positioning of insert
xki1-3'R-trans	AGCCTAAAGTTGCCTGTTTTCGG	Positioning of insert
hph-5'R-trans	CGCTCGTCTGGCTAAGATCG	Positioning of insert
hph-3'F-trans	AAGGAATCGGTCAATACACTACATGG	Positioning of insert
Probe-hph-F	ATGAAAAAGCCTGAACTCACCGC	Amplification of DIG probe
Probe-hph-R	TATTCCTTTGCCCTCGGACG	Amplification of DIG probe
