# Supplementary material for: Deletion of either the regulatory gene ara1 or metabolic gene xki1 in Trichoderma reesei leads to increased CAZyme gene expression on crude plant biomass
Source: Biotechnol Biofuels. 2019 Apr 9;12:81. doi: 10.1186/s13068-019-1422-y (PMC6454604; doi:10.1186/s13068-019-1422-y)
Supplement: Supplementary file 5 — Additional file 5. Pearson correlation matrix of Trichoderma reesei transcriptomes. Raw gene counts were used to evaluate the level of correlation between biological replicates using Pearson’s correlation. Pearson correlation matrix were performed in R (v3.4.0) statistical language and environment, the core function from the stats base package and the corrplot (v 0.77) package were used for the analysis. One sample (reference strain on SBH at 48 h) was removed from the dataset because it correlated poorly with its replicates. [file 13068_2019_1422_MOESM5_ESM.pdf]

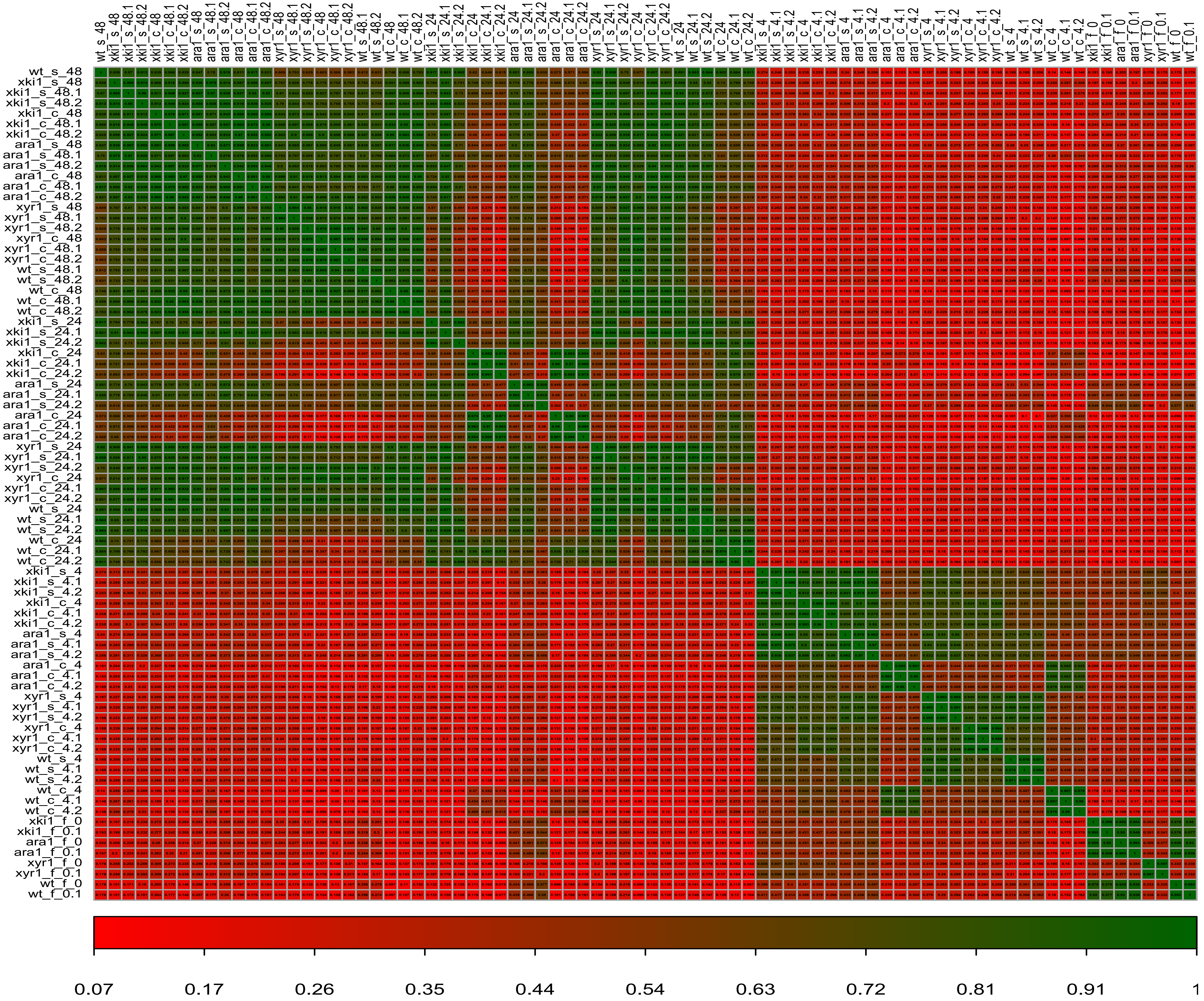

Additional file 5. Pearson correlation matrix of *T. reesei* transcriptomes.

Raw gene counts were used to evaluate the level of correlation between biological replicates using Pearson's correlation. Pearson correlation matrix were performed in R (v3.4.0) statistical language and environment, the cor function from the stats base package and the corrplot (v 0.77) package were used for the analysis. One sample (wt on SBH at 48h) was removed from the dataset because it correlated poorly with its replicates.
