# Supplementary material for: Deletion of either the regulatory gene ara1 or metabolic gene xki1 in Trichoderma reesei leads to increased CAZyme gene expression on crude plant biomass
Source: Biotechnol Biofuels. 2019 Apr 9;12:81. doi: 10.1186/s13068-019-1422-y (PMC6454604; doi:10.1186/s13068-019-1422-y)
Supplement: Supplementary file 6 — Additional file 6. Analysis of the protein banding patterns from the three replicate culture supernatants of T. reesei reference (ref) and Δxyr1 strains cultured with either corn stover or soybean hulls for 24 h and 48 h. The same volume of culture supernatant was loaded for all samples. [file 13068_2019_1422_MOESM6_ESM.pdf]

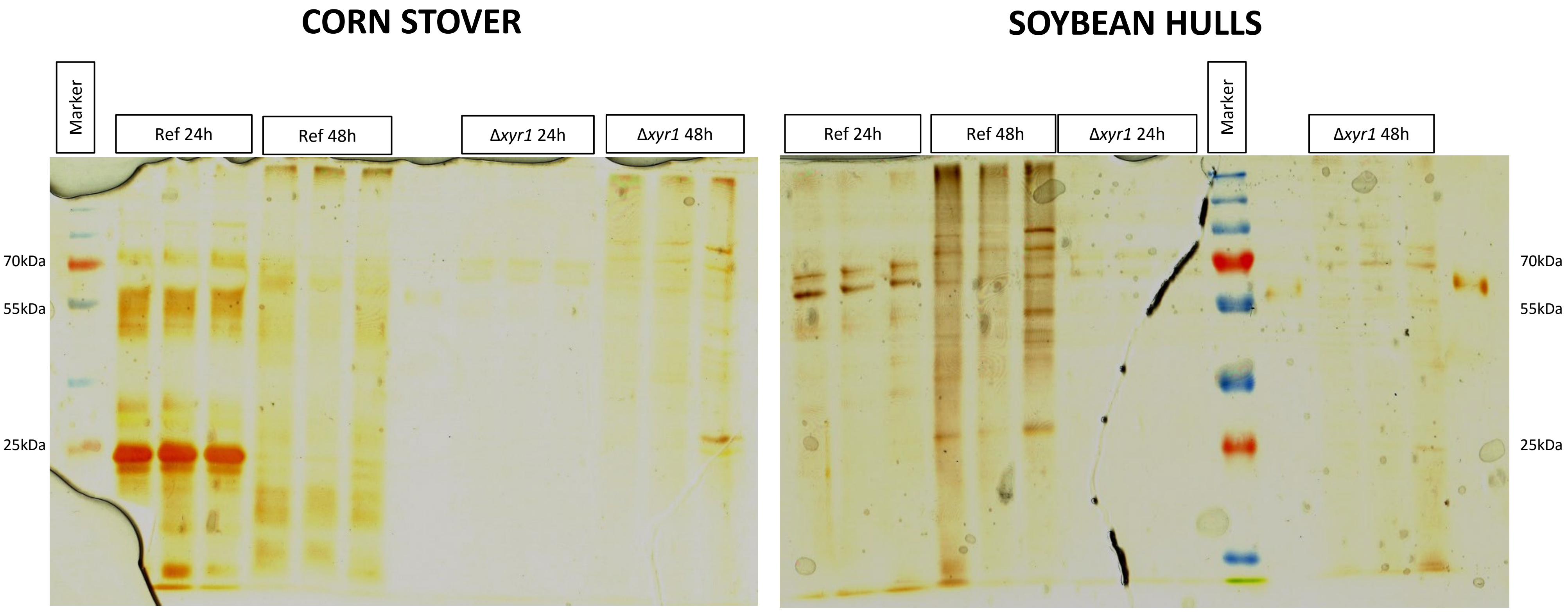

Additional file 6. Analysis of the protein banding pattterns from the three replicate culture supernatants of *T. reesei* reference (ref) and **Δ***xyr1* strains cultured with either corn stover or soybean hulls for 24h and 48h. The same volume of culture supernatant was loaded for all samples.
